# Quantitative Assessment of 3D Printed Model Accuracy in Delineating Congenital Heart Disease

**DOI:** 10.3390/biom11020270

**Published:** 2021-02-12

**Authors:** Shenyuan Lee, Andrew Squelch, Zhonghua Sun

**Affiliations:** 1 Discipline of Medical Radiation Science, Curtin Medical School, Curtin University, GPO Box, U1987, Perth, WA 6845, Australia; shen-yuan.lee@postgrad.curtin.edu.au; 2 Discipline of Exploration Geophysics, Western Australian School of Mines, Minerals, Energy and Chemical Engineering, Curtin University, Perth, WA 6845, Australia; a.squelch@curtin.edu.au

**Keywords:** 3D printing, congenital heart disease, model, simulation, accuracy, comparison

## Abstract

Background: Three-dimensional (3D) printing is promising in medical applications, especially presurgical planning and the simulation of congenital heart disease (CHD). Thus, it is clinically important to generate highly accurate 3D-printed models in replicating cardiac anatomy and defects. The present study aimed to investigate the accuracy of the 3D-printed CHD model by comparing them with computed tomography (CT) images and standard tessellation language (STL) files. Methods: Three models were printed, comprising different CHD pathologies, including the tetralogy of Fallot (ToF), ventricular septal defect (VSD) and double-outlet right-ventricle (DORV). The ten anatomical locations were measured in each comparison. Pearson’s correlation coefficient, Bland–Altman analysis and intra-class correlation coefficient (ICC) determined the model accuracy. Results: All measurements with three printed models showed a strong correlation (r = 0.99) and excellent reliability (ICC = 0.97) when compared to original CT images, CT images of the 3D-printed models, STL files and 3D-printed CHD models. Conclusion: This study demonstrated the high accuracy of 3D-printed heart models with excellent correlation and reliability when compared to multiple source data. Further investigation into 3D printing in CHD should focus on the clinical value and the benefits to patients.

## 1. Introduction

Congenital heart disease (CHD) is the most common congenital abnormality, responsible for high infant mortality globally [1,2]. It is accompanied by deformities and complex structures that make the surgical planning and treatment for complicated CHD challenging for paediatric cardiologists [3]. At present, the diagnosis of CHD is mainly based on two-dimensional (2D) images, using computed tomography (CT), magnetic resonance imaging (MRI) or echocardiography imaging; however, it is difficult to present the complex structure of CHD on traditional 2D or three-dimensional (3D) visualisation due to the wide variability of the pathologies [4]. This limitation of the method is overcome with 3D-printing technology, which has increasing applications in the medical field [5,6,7].

3D-printing in medical applications is expanding rapidly for children and has shown a promising role in overcoming the limitations of traditional image visualisations, especially the CHD. Currently, 3D-printed models are mainly used in three areas: presurgical planning and simulation, medical education and communication with patient and family [8,9,10,11,12,13,14,15,16,17,18]. The main advantage of the 3D-printed heart model created from CT and MRI images and echocardiography imaging is that it can demonstrate a realistic 3D spatial correlation between the heart and the surrounding structures, which is difficult to obtain from conventional 2D and 3D visualisations [19,20]. Despite the model accuracy of depicting cardiac anatomy and pathology based on isolated case reports or case series, there is a lack of studies validating the model accuracy when compared to standard tessellation language (STL) files and CT-scanned model images [21]. A systematic review showed that out of seven studies that investigated the model accuracy of CHD, only four reported the mean bias and standard deviations of measurements of model dimensional accuracy, and the remaining three only reported the correlation coefficient between 3D-printed models and measurements [22].

Although the high accuracy of the 3D-printed CHD models has been achieved with current studies when compared to the original image data, there is no standard reference to confirm the acceptable range of error for highly accurate 3D-printed models. The majority of the studies available in the literature reported a model accuracy based on the comparison of original source images with 3D-printed models, while there is no report on the comparison of a segmented volumetric surface STL file with 3D-printed models regarding dimensional accuracy. The generation of an STL file is a critical step for heart model printing with respect to data post-processing and segmentation. These image post-processing and segmentation processes, as well as technical aspects of 3D printing, can all affect the accuracy of 3D-printed models. Hence, the model accuracy needs to be validated by comparing with original images and segmentation data (STL). Therefore, the present study aimed to determine the accuracy of the 3D-printed model by evaluating every step involved in creating the models. This included a series of comparisons between original CT images, 3D-printed model, CT of 3D-printed models and STL files in terms of model accuracy. Consequently, slight differences were detected, as expected, between the different stages of model creation. One of the validation methods used in this study was to CT scan 3D-printed models and compare them with original source imaging data and segmented files for the determination of model accuracy. This represents an innovative aspect of this study, as it comprises a comprehensive assessment of all aspects that are involved in generating the 3D-printed heart models.

## 2. Materials and Methods

### 2.1. CT Data Collection

Ethical approval was obtained from the Curtin Human Research Ethics Committee. The cardiac computed tomography angiography (CCTA) images of complex CHD in six cases were collected anonymously in the digital imaging and communications in medicine (DICOM) format. These CHD cases included two of tetralogy of Fallot (ToF), two of the ventricular septal defect (VSD) and two of double-outlet right-ventricle (DORV). The CT scans were performed either on a 256-slice GE Revolution scanner (Revolution CT, GE Healthcare, Waukesha, WI, USA) or a 128-slice Siemens Definition Flash (Somatom Definition Flash, Siemens Healthcare, Forchheim, Germany) using the following settings: 70 kilovoltage peak (kVp), 120 milliampere seconds (mAs) and slice thickness 0.5 mm.

### 2.2. Image Segmentation and Post-Processing

**Figure 1:**
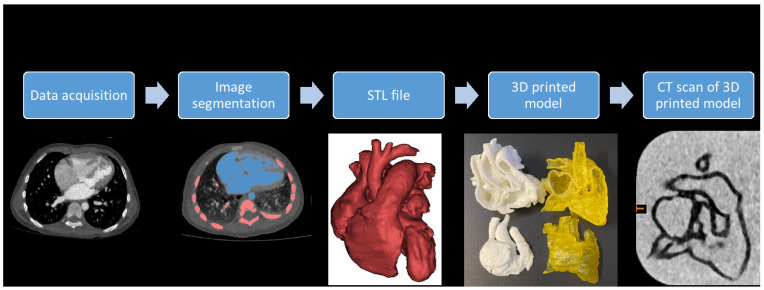
The steps involved in image post-processing and segmentation for creation of a 3D-printed heart model.

Mimics Innovation Suite 22.0 (Materialise HQ, Leuven, Belgium) was used for image post-processing and segmentation in all six cases. Figure 1 shows the steps of creating a 3D-printed heart model. DICOM images were transferred into Mimics for image segmentation based on thresholding and multi-slice editing. Thresholding isolates voxels, with attenuation within a specified Hounsfield unit (HU) range, by choosing different threshold ranges that can create multiple masks to represent various anatomical structures. The blood pool of the heart was isolated from other anatomical structures, and 2 mm layer thickness was added outside the blood pool to facilitate printing of the heart model.

3-Matic software (Materialise HQ) was used for fixing holes and smoothing the digital file by the wrapping and smoothing function. The STL file was exported to a mesh mixer from 3-Matic for further editing. The 3D model was dissected into a two-piece component for viewing the internal structure of the heart, especially a distinct visualisation and examination of the defect of each model.

### 2.3. 3D Printing of Heart Models

Two printers were used in this study for printing patient-specific heart models. The first printer was an Ultimaker 2 Extended 3D printer (Ultimaker BV, Geldermalsen, The Netherlands) with a build volume of 223 × 223 × 305 mm^3^. The XY resolution of the Ultimaker 2 printer is 12.5 µm, while Z resolution depends on the nozzle size. The nozzle size used in printing heart models in this study was 0.4 mm, with a z resolution of 100 µm. The printing material was fused filament fabrication (FFF) in thermoplastic polyurethane (TPU) 50 A. Each model was printed for approximately 31 h.

The second printer was Anycubic Photon S (Anycubic, Shenzhen, Guangdong, China) using liquid crystal display (LCD)-based stereolithography (SLA) with printing resolution of 50 µm. The models were printed using the following materials: Magma H LINE Photopolymer Resin (Magma Filament, Kuala Lumpur, Malaysia) and Monocure Flex100 Rapid Resin (Monocure, New South Wales, Australia), and the printing time was about 19 h.

**Figure 2:**
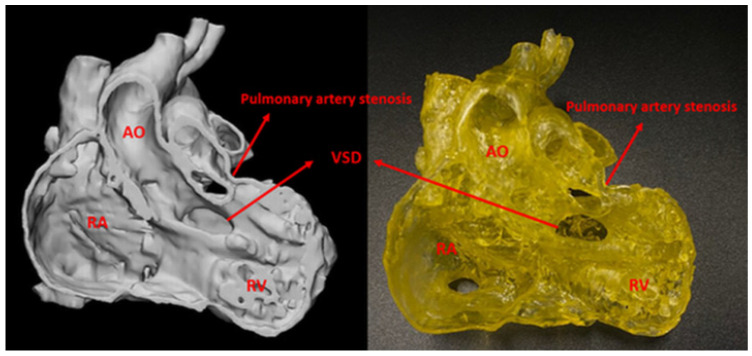
Comparison between digital file (left) and 3D-printed model (right). AO, aorta; VSD, ventricular septal defect; RV, right ventricle; RA, right atrium.

Three cases with good image quality were selected for printing. The first two cases were patients with VSD and DORV (Figure 2), printed in a yellow colour using Magma H LINE Photopolymer Resin and Monocure Flex100 Rapid Resin. The third case is a patient with ToF, and the model was printed with TPU-50A in white colour.

### 2.4. 3D Model Accuracy Assessment

**Figure 3:**
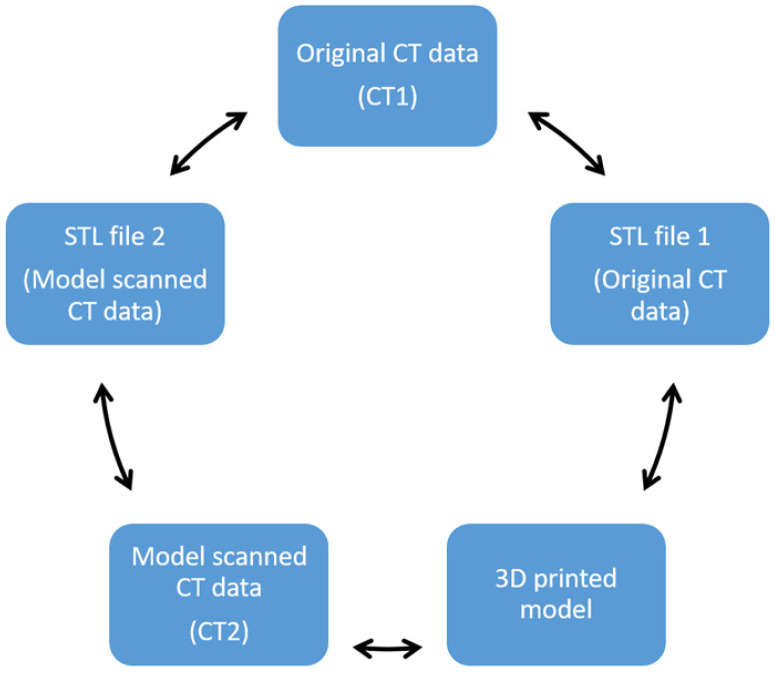
Multiple comparisons were performed to investigate the accuracy of the 3D-printed model.

A total of five comparisons, involving CT image data, STL file and a 3D-printed model, were performed as shown in Figure 3. Subsequently, ten anatomical locations were chosen to measure in the five different components (Table 1). The maximal transverse and longitudinal dimensions of every anatomical location were measured three times with the mean value considered as final to reduce the bias. Two assessors performed these measurements independently and inter-rate reliability was assessed by intraclass correlation coefficient.

**Figure 4:**
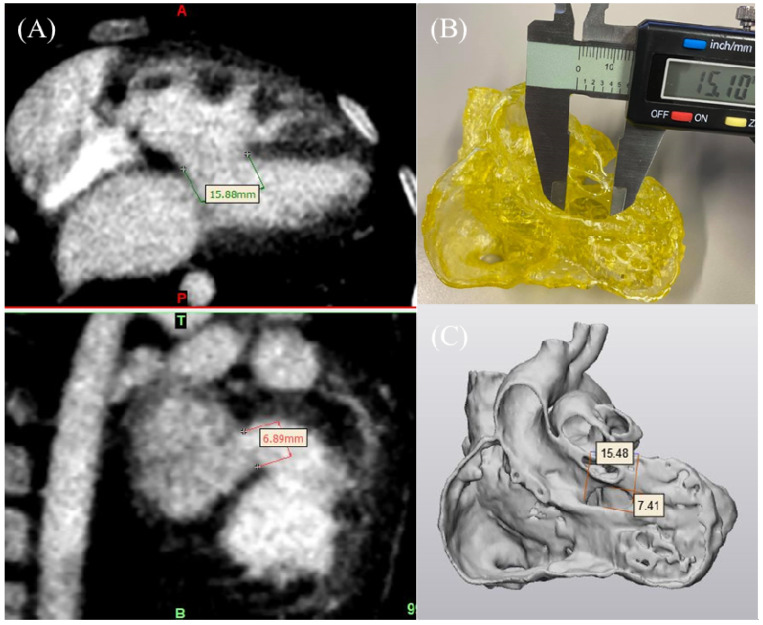
3D model accuracy evaluation. (**A**) CT imaging data in coronal and sagittal views to measure the VSD in Mimics. (**B**) Measurement of the VSD in the 3D-printed model using a digital calliper. (**C**) STL file measurement of the VSD in 3-Matic. VSD-ventricular septal defect.

DICOM images were transferred into Mimics using the ruler function for measurements. STL files were transferred into 3-Matic using the distance function. The 3D-printed model was evaluated using an electronic calliper, as shown in Figure 4. The 3D-printed models were scanned using a 192-slice Siemens scanner (Siemens Force, Siemens Healthcare, Forchheim, Germany) under the following imaging protocols: slice thickness 0.5 mm, gantry rotation time 0.25 s, FoV 250 mm and 80 kVp. During scanning, the non-ionic contrast medium Omnipaque 300 mgI/mL (IOHEOL 32.35 g/50 mL) was used at 6–8% dilution to create CT attenuation similar to that used in routine cardiac CT angiography (about 400 HU) to increase the visibility of the heart chamber. The models were placed in a plastic container while scanning with the contrast medium.

### 2.5. Statistical Analysis

SPSS version 26.0 (IBM Co., Armonk, NY, USA) was used for the statistical analyses. Pearson’s correlation was used to assess the correlation between every two measurements. Bland–Altman analysis was used to describe the agreement between two measurements. Intraclass correlation coefficient (ICC) was used to assess the reliability between measurements of the five components. A *p*-value < 0.05 indicated statistical significance.

## 3. Results

Three CHD heart models were printed in this study, including a 7-month-old boy with VSD, 17-month-old boy with DORV and 3-month-old girl with ToF. Model segmentations needed 2–2.5 h in the beginning, and after becoming accustomed to the software, this duration reduced to 1–1.5 h. The cost of printing for each heart model was approximately 50 AUD.

**Figure 5:**
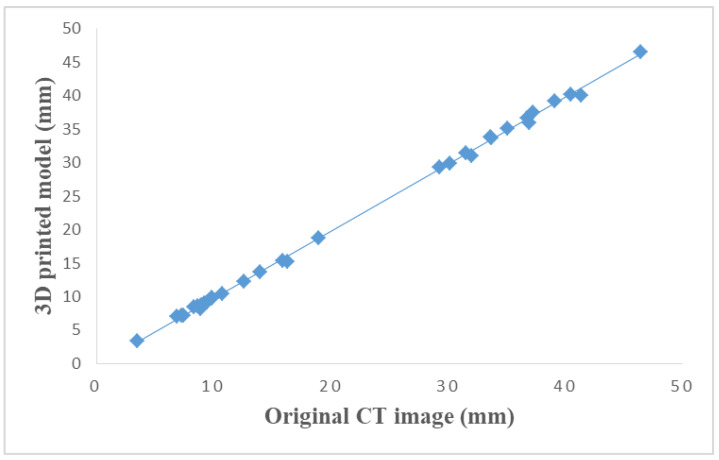
Scatterplot between original CT measurements and 3D-printed model measurements.

A total of 33 vascular diameters were measured in these five components. A strong correlation (r = 0.99) with statistically significant linear correlation (*p* < 0.001) was found in all comparisons. The differences between each comparison are shown in Table 2. Figure 5 demonstrates the measurements of original CT data and 3D-printed model with an excellent correlation (r = 0.99).

**Figure 6:**
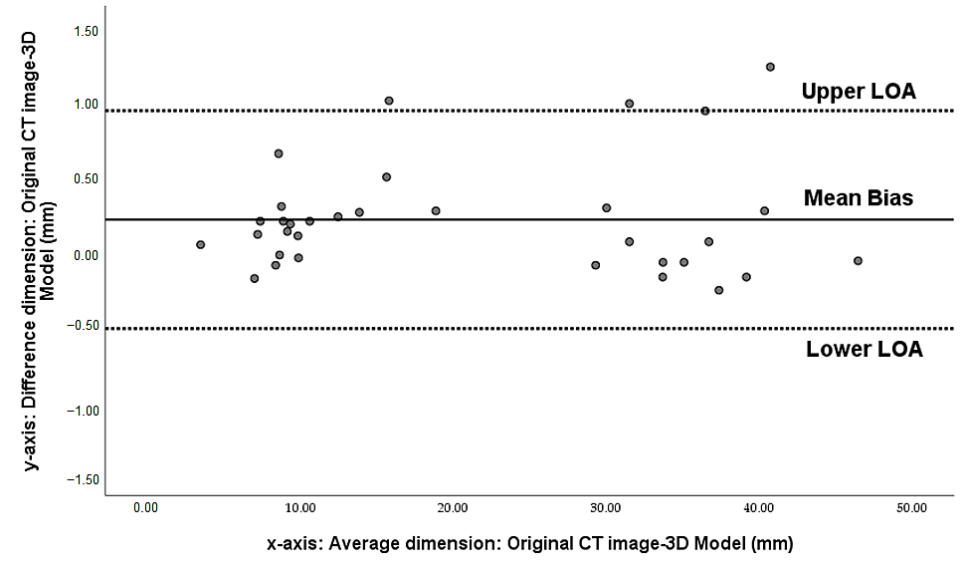
3D printed model dimension accuracy. Bland-Altman plot measurement agreements between original CT image and 3D-printed model.The limits of agreement (LOA) are calculated as the mean difference ± 1.96 Std Dev.

The Bland–Altman analysis confirmed the accuracy of the 3D-printed model based on a high correlation of dimensional measurements, and the slope of the regression line was not significant between every comparison. The mean difference between original CT data (CT1) and 3D-printed model was 0.21 ± 0.37 mm, indicating that the accuracy of the 3D model was <0.5 mm. Similarly, the difference in measurements between the CT scan of the 3D-printed model and the other four variables was <0.5 mm. Figure 6 is an example of a Bland–Altman plot comparing original CT data with those of the 3D-printed model. Excellent reliability was found among all measurements, and the average ICC was 0.97.

## 4. Discussion

In the present study, we compared every measurement, including the original source CT images, STL files, 3D-printed models and CT scan of the 3D-printed models, involved in each step of creating the 3D-printed CHD model, and recorded the mean bias and standard deviations in order to determine the model accuracy in delineating cardiac anatomical structures and pathologies. The difference between these comparisons was <0.5 mm, with a percentage difference between 0.4 and 0.9%. All the measurements were compared using Pearson’s correlation (r = 0.99), Bland–Altman analysis and ICC (ICC = 0.97, *p* < 0.001). The results showed that none of the measurements showed a significant difference but had a high correlation and reliability. Thus, this study further validated the accuracy of 3D-printed heart models.

Table 3 is a summary of the current studies in the literature, reporting a 3D-printed heart model accuracy with a mean difference and standard deviation. In the study by Valverde et al., conducted across ten different international centres, 20 cases with original CT/MRI image data and a 3D-printed model were compared using the Bland–Altman analysis; the difference was 0.27 ± 0.73 mm [12]. Olejník et al. included eight CHD cases and compared 3D heart models and digital images using Bland–Altman analysis, and the difference was 0.19 ± 0.38 mm [23]. The mean absolute error in the measurements from nine patients comparing 3D echocardiography and a 3D-printed model was 0.4 ± 0.9 mm in a study by Olivieri et al. [24], demonstrating the high accuracy of 3D-printed heart models and established an excellent correlation between 3D echocardiographic images and models. Furthermore, Lau et al. reported the mean difference between the measurements of the 3D-printed CHD model and the original CT image, which was 0.23 mm based on the analysis of a single case [25]. Mowers et al. compared a 2D echo with digital 3D models and 2D echo with printed 3D models, and showed a difference of 0.0 mm and 0.3 mm, respectively [26]. Other studies showed a strong correlation between 3D models and digital images but did not report the mean differences [27,28,29,30].

In the current study, we compared digital image data, STL files and 3D-printed models as well as the CT scan of the 3D-printed models. When comparing the 3D printed model with STL files created from the original CT data (STL1) and original CT data, the mean difference is 0.1 ± 0.28 and 0.21 ± 0.37 mm, respectively. Furthermore, the 3D printed model was scanned using CT to obtain another set of imaging data (CT2) and created another STL file (STL2) from the CT2 for accuracy validation. The mean difference in the comparison of the original CT data with CT2 was 0.1 ± 0.4 mm, while that of STL1 with STL2 was 0.1 ± 0.45 mm. To the best of our knowledge, this is the first study comparing the model accuracy with STL file and CT scan of the models, as most of the other studies focused on comparing the 3D-printed models with original source images, while the STL file is a critical step for 3D-printed models and should be included in the comparisons. Moreover, our results add valuable information to the current literature regarding model accuracy in delineating cardiac structures and pathologies.

The major element of a 3D-printed CHD model is its accuracy, especially in presurgical planning and preoperative simulation. The 3D-printed CHD models have been primarily used for surgical planning, surgical simulation and medical education. The review by Illmann et al. indicated that a 3D-printed CHD model could serve as the optimal device for treatment and location. It can also demonstrate the complexity of the intra-cardiac structures and changes in the treatment plans. However, the model accuracy was not discussed [31].

In a systematic review, Lau and Sun put forth two reasons that could impact the accuracy of the printed model: image acquisition technique and image resolution. Among 28 studies investigating the use of CHD model in their systematic review, only six studies reported the quantitative assessment of the printed CHD model accuracy [32]. In another recent systematic review and meta-analysis of 24 studies, only three studies established a correlation between the printed CHD model and imaging data, while four studies recorded the mean difference and standard deviation of model accuracy measurements [22]. Batteux et al. analysed 11 studies on the 3D-printed model’s accuracy and showed that a variety of segmentation software tools, statistical methods and data for accuracy comparison used in these studies, resulting in heterogeneous approaches that are utilised to measure model accuracy. A satisfactory accuracy of the 3D-printed models was achieved despite methodological heterogeneity [33]. Accuracy is vital for clinical application of the CHD model, especially in presurgical planning and treatment as an accurate representation of patient anatomy and pathology by 3D-printed models guarantees the successful performance of surgical procedures. Thus, additional studies with more printed CHD models should be carried out to establish an optimal range of error for the model.

The current literature review revealed that the 3D-printed models are highly accurate, and the error was <0.5 mm. However, for the study of the quantitative analysis of the CHD model accuracy, not all the studies compared the model with the original source images. Studies investigating model accuracy with mean bias and standard deviation and comparing the measurements with the STL file are still lacking. There is no available acceptable level of difference in 3D-printed heart models, although the 0.5% tolerance level was reported for 3D-printed brain models [34]. Many factors could affect the dimensional accuracy of 3D-printed models including source data quality, image post-processing and segmentation steps for creation of virtual models and 3D printing resolution [35]. Of these factors, the resolution of 3D printers has a direct impact on model accuracy. Carvalho et al., in their recent experimental study, developed 3D-printed biomodels simulating coronary stenosis and investigated the relationship between printing resolutions and hemodynamic flow changes [36]. Their analysis showed that the model printed with 50 µm resolution had the lowest error of 10% compared to higher errors of 28% and 50%, with models printed with 100 and 150 µm resolution, respectively. Of the ten comparisons in our study, as shown in Table 2, a 0.5% percentage difference was found in six of them, indicating the high accuracy or reliability of our printed models. This could be due to the high printing resolution of from 50 to 100 µm in our study. Hitherto, this is the only study that has addressed the gap in the existing literature and confirms the model accuracy, despite complex image post-processing and segmentation processes. The findings of this study could be used to guide further research on determining the accuracy of 3D-printed heart models.

Nevertheless, one of the limitations of this study was the inclusion of only three models. For the printed model from paediatric CT images, heart-rate-control could be challenging, which might impact the quality of the CT images and make image segmentation and post-processing more difficult compared to that with adults’ imaging data. It is also difficult to print the location for a heart valve. The simulation of heart beats during CT scan will represent realistic physiological situation for cardiac CT imaging; unfortunately, this was not addressed in this study since our focus was to determine model accuracy through the comparison of different data files. Hemodynamic analysis of the 3D-printed heart models/chambers could be simulated in future studies to address this limitation. Another limitation is the lack of validation of the clinical value of 3D-printed heart models, as the focus of the current study was to determine the model accuracy rather than investigating its diagnostic value. This limitation could be addressed in future studies.

## 5. Conclusions

In conclusion, the present study showed that highly accurate heart models could be printed with excellent intracardiac structures and cardiac defects. Quantitative analysis demonstrated very minor differences between 3D-printed models and original CT images and segmented STL files, further highlighting the accuracy of 3D models in delineating cardiac anatomy and pathology. Finally, the use of 3D printing in congenital heart disease should focus on the clinical application of 3D-printed personalised models in improving patient outcomes.

## Figures and Tables

**Table 1:** Anatomical locations for measurement comparisons.

	Anatomical Locations
1	Right atrium
2	Right ventricle
3	Left atrium
4	Left ventricle
5	Aortic arch
6	Ascending aorta
7	Descending aorta
8	Pulmonary trunk
9	Superior vena cava
10	Ventricular septal defect

**Table 2:** Difference between each comparison of model accuracy measurements.

Comparison	Difference (mm) (mean ± SD)	Percentage Difference (%)
CT1 vs. STL1	0.12 ± 0.23	0.5 ± 1.06
STL1 vs. 3D model	0.1 ± 0.28	0.4 ± 1.32
3D model vs. CT2	−0.11 ± 0.47	0.5 ± 2.24
CT2 vs. STL2	0.12 ± 0.25	0.6 ± 1.18
STL2 vs. CT1	−0.23 ± 0.47	0.9 ± 2.24
CT1 vs. 3D model	0.21 ± 0.37	0.9 ± 1.7
CT1 vs. CT2	0.1 ± 0.40	0.5 ± 1.8
STL1 vs. CT2	−0.12 ± 0.42	0.5 ± 1.9
STL1 vs. STL2	0.1 ± 0.45	0.4 ± 2.13
3D model vs. STL2	0.17 ± 0.48	0.8 ± 2.28

**Table 3:** Measurements for 3D-printed CHD models with mean difference according to the current literature.

Studies Reporting Accuracy	No. of Models Printed	Comparisons	Mean Difference (mm)	Analysis Method
Valverde et al. [12]	20	3D model vs. CT/MRI	0.27 ± 0.73 mm	Pearson
Olejník et al. [23]	8	3D model vs. DICOM	0.19 ± 0.38 mm	Bland–Altman
		3D model vs. vivo	0.13 ± 0.26 mm	
Olivieri et al. [24]	9	3D model vs. Echo	0.4 ± 0.9 mm	Pearson/Bland–Altman
Lau et al. [25]	1	3D model vs. CT	0.23 mm	Pearson
Mowers et al. [26]	5	2D echo vs. digital 3D	0 mm	Pearson/Bland–Altman
		2D echo vs. 3D model	0.3 mm	
Our study	3	3D model vs. original CT/CT of 3D model	0.21 ± 0.37/ −0.11 ± 0.47 mm 0.1 ± 0.28/0.17 ± 0.48 mm 0.12 ± 0.23/0.12 ± 0.25 mm	Pearson/Bland–Altman
		3D model vs. STL files		
		CT images vs. STL files		

## Data Availability

No new data were created or analyzed in this study. Data sharing is not applicable to this article.
